# Genomics‐led approach to drug testing in models of undifferentiated pleomorphic sarcoma

**DOI:** 10.1002/1878-0261.70059

**Published:** 2025-05-26

**Authors:** Piotr J. Manasterski, Molly R. Danks, John P. Thomson, Morwenna Muir, Martin Lee, John C. Dawson, Ana T. Amaral, Juan Diaz‐Martin, David S. Moura, Javier Martin‐Broto, Ali Alsaadi, Donald M. Salter, Ailsa J. Oswald, Graeme Grimes, Larry Hayward, Ted R. Hupp, Karen Sisley, Paul H. Huang, Neil O. Carragher, Valerie G. Brunton

**Affiliations:** ^1^ Cancer Research UK Scotland Centre (Edinburgh), Institute of Genetics and Cancer University of Edinburgh UK; ^2^ Instituto de Biomedicina de Sevilla, IBiS/Hospital Universitario Virgen del Rocío/CSIC/Universidad de Sevilla Spain; ^3^ Tumor Microenvironment and Targeted Therapies Group, CiBB‐Center for Innovation in Biomedicine and Biotechnology, Universidade de Coimbra Portugal; ^4^ Research Health Institute of Fundacion Jimenez Diaz (IIS/FJD; UAM) Madrid Spain; ^5^ Department of Medical Oncology Fundacion Jimenez Diaz University Hospital Madrid Spain; ^6^ University Hospital General of Villalba Madrid Spain; ^7^ Centre for Genomics & Experimental Medicine, Institute of Genetics and Cancer University of Edinburgh UK; ^8^ MRC Human Genetics Unit, Institute of Genetics and Cancer University of Edinburgh UK; ^9^ Division of Clinical Medicine, School of Medicine and Population Health, The Medical School University of Sheffield UK; ^10^ Division of Molecular Pathology The Institute of Cancer Research London UK; ^11^ Present address: Dubai Genomic Center Al Jalila Children Hospital Dubai United Arab Emirates

**Keywords:** drug screening, patient‐derived xenograft, tumour slices, undifferentiated pleomorphic sarcoma, whole exome sequencing

## Abstract

Undifferentiated pleomorphic sarcoma (UPS) is a rare cancer with limited systemic treatment options and poor outcomes. To seek novel therapeutic interventions, we undertook mutational analysis of 20 UPS patient tumours, four established UPS cell lines and three patient‐derived xenograft (PDX) models. Frequently mutated genes were uncommon; in contrast, copy number (CN) events were common with CN gain frequently observed at genes including *JUN*, *EGFR* and *CDK6* and loss at *WNT8B*, *RB1* and *PTEN*. Analysis of overlapping genomic changes between patient tumours and PDX models or cell lines revealed druggable events. A selected panel of drugs targeting these was analysed in *in vitro* UPS models demonstrating that the *mitogen‐activated protein kinase kinase* (MEK) inhibitor trametinib is synergistic in combination with the fibroblast growth factor receptor (FGFR) inhibitor infigratinib. This was further confirmed to be efficacious in an *ex vivo* tumour slice model. Taken together, our results demonstrate the rationale for utilising genomic data to identify drug classes targeting druggable events in low‐prevalence cancers and indicate that trametinib alone or in combination with infigratinib should be further explored for clinical UPS management.

## GA text

Genomic data from undifferentiated pleomorphic sarcoma patients and preclinical models were used to inform a targeted drug screen. Selected compounds were tested in 2D and 3D cultures of UPS cell lines. A combination of trametinib and infigratinib was synergistic in the majority of UPS cell lines tested, which was further confirmed in an *ex vivo* tumour slice model.

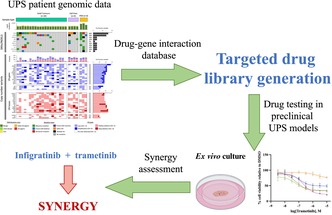

**Abbreviations**
CDK: cyclin‐dependent kinase
CN: copy number
FGFR: fibroblast growth factor receptor
HCO: high‐confidence oncogenic
INDELS: short insertions and deletions
JAK: Janus kinase
MEK: *mitogen‐activated protein kinase kinase*
PARP: poly (ADP‐ribose) polymerase
PCA: principal component analysis
PDX: patient‐derived xenograft
PI3K: phosphatidylinositol 3‐kinase
RPPA: reverse‐phase protein array
SNVs: single nucleotide variants
STS: soft tissue sarcoma
TMB: tumour mutational burden
UPS: undifferentiated pleomorphic sarcoma
VEGFR: vascular endothelial growth factor receptor
VUS: variants of unknown significance
WES: whole exome sequencing

## 1 Introduction

Soft tissue sarcomas (STSs) are a heterogeneous group of tumours of mesenchymal origin that account for approximately 1% of all cancers [1]. UPS, formerly referred to as malignant fibrous histiocytoma, accounts for approximately 10–20% of all STS cases and is comprised of tumours with no identifiable line of differentiation [2]. UPS is characterised by poor 5‐year survival rates of 30–50% following diagnosis and an approximately 40% metastasis rate [3, 4, 5]. The clinical management of UPS is centred around margin‐negative surgical resection with or without adjuvant radiotherapy and systemic chemotherapy [6, 7]. Despite the response rate to single‐agent treatment being low, doxorubicin remains the first‐line therapy, followed by ifosfamide as second‐line treatment [7, 8]. As patients with relapsed high‐grade UPS and those who present with advanced disease have a poor prognosis, there is a need for novel approaches that will improve outcomes.

UPS are characterised by complex karyotypes and a lack of well‐defined genetic driver events, and their heterogeneity is characterised by CN variations rather than single point mutations [9, 10, 11, 12, 13, 14]. Nevertheless, recurring mutations in a selected number of genes have been identified. *TP53* was identified as the most commonly mutated gene in UPS patients with the prevalence reported to be as high as 69% [10, 12, 13, 14, 15, 16]. Frequent mutations in *ATRX*, *RB1*, *PTEN*, *CDKN2A* and *KMT2C* have also been reported [10, 12, 13, 14, 17]. Gene fusion events involving *RB1* and *PRDM10* are present in a small subset of UPS cases, although their functional significance is unclear [9, 13, 16, 18]. Although genomic analysis of UPS has uncovered potentially clinically actionable alterations, including a case report where this approach led to the identification of an efficacious therapy in a mouse PDX model [19], there are no reports of this leading to changes in clinical treatment decisions or the recruitment of patients into clinical trials, in common with other STS subtypes [13, 20].

Another limiting factor in the identification of effective treatments for UPS has been the lack of preclinical models that reflect the clinical disease which can be used to support the evaluation of potential new drugs. Here we have used a panel of well‐annotated UPS cell lines [21, 22] and describe the generation of a novel PDX‐derived UPS cell line, showing that their genomic profiles mirror those seen in patient samples. We also demonstrate the ability of PDX‐derived tumour slices to provide an alternative *ex vivo* approach to assess drug responses. The genomic data was used to identify druggable events and potentially efficacious drug classes which were then tested in our preclinical UPS models. *In vitro* and *ex vivo* results indicate that the MEK inhibitor trametinib, in combination with the FGFR inhibitor infigratinib, should be investigated further as an alternative or complementary treatment to currently approved UPS therapies.

## 2 Materials and methods

### 2.1 Ethics and clinical samples

The study was conducted according to the guidelines of general approval for use of surgically obtained tissue and approved by NHS Lothian NRS BioResource and the Public Health Office with the understanding and written consent of each subject and conforming to the standards set by the Declaration of Helsinki. The Lothian NRS BioResource is an HRA‐approved research tissue bank (REC Ref: 20/ES/0061). Approval was given by East of Scotland Research Ethics Service REC 1. Twenty UPS samples and adjacent normal tissue were collected at surgery. Before exome sequencing, H&E‐stained sections from each sample were evaluated by a pathologist (D.M.S.) and verified as UPS.

### 2.2 PDX models

The comparison of morphology and vimentin, cytokeratin and Ki67 staining between the original patient tumours and IEC‐16 and IEC‐56 PDX models is shown in Fig. S1. IEC‐56 was propagated by bilateral subcutaneous implantation of tumour fragments into 6–8‐week‐old CD‐1 Nude (Charles River, Wilmington, MA, USA) and Rag2‐Il2rg double knock‐out (R2G2; Inotiv, Lafayette, IN, USA) female mice. Mice were monitored twice weekly and tumour length and width were measured using a calliper; tumour volume was calculated using the formula ([*L* × *W* ^2^]/2). Upon reaching a 10 mm tumour diameter, mice were culled, and a portion of each tumour was removed and stored in 10% NBF solution (Sigma‐Aldrich, Saint Louis, MO, USA) for embedding. The remaining tumour was stored in PBS for tissue slicing or culture, re‐implanted fresh or frozen as fragments for passage. All animal experiments were approved by the University of Edinburgh Animal Welfare and Ethical Review Body (PL05‐21) and carried out according to the guidelines set by the UK Home Office Regulations (Animals [Scientific Procedures] Act 1986) under licence number PP7510272.

### 2.3 PDX tumour slicing and culture

The Compresstome™ VF‐310‐0Z (Precisionary Instruments; speed setting: 5, oscillation setting: 9) was used to obtain precision‐cut tissue sections (300 μm) from IEC‐56 PDX tumours were embedded in 2% agarose gel (Invitrogen, Waltham, MA, USA). Tissue slices were cultured on top of 0.4 μm Millicell cell culture inserts (Millipore, Burlington, MA, USA) in six‐well plates. The slices were cultured in Advanced DMEM (Gibco, Waltham, MA, USA) supplemented with 10% v/v FBS (Gibco), 1% v/v Penicillin–Streptomycin (Gibco) and 1% v/v GlutaMAX (Gibco).

### 2.4 Generation of IEC‐56 PDX‐derived cell line

Fresh IEC‐56 PDX tumours were minced into small fragments and placed in a shaking incubator at 37 °C for 1 h in dissociation medium containing Advanced DMEM (Gibco), 5% v/v FBS, 1% v/v Penicillin–Streptomycin, 1% v/v ITS (Gibco), 10 ng·mL^−1^ EGF (PeproTech, Cranbury, NJ, USA), 10 μg·mL^−1^ Hydrocortisone (Sigma‐Aldrich), 0.5 mg·mL^−1^ Collagenase (Sigma‐Aldrich), 0.1 mg·mL^−1^ Hyaluronidase (Sigma‐Aldrich) and 0.1 mg·mL^−1^ DNase I (Sigma‐Aldrich). The cell suspension was subsequently incubated in RBC Lysis Buffer (Invitrogen), followed by incubations with 0.05% Trypsin/EDTA and 1 mg·mL^−1^ DNase I before passing through a 70 μm filter. All reagents were supplemented with 10 μM Y27632 dihydrochloride (Tocris, Bristol, UK). The mouse cells were separated from the suspension by magnetic‐activated cell sorting using the Mouse Cell Depletion Cocktail and LS Columns (Miltenyi Biotec, Bergisch Gladbach, Germany) according to the manufacturer's instructions. For cell line generation, the cells were seeded in a 10 cm cell culture dish left undisturbed for 5–7 days before any medium changes.

### 2.5 Cell culture

The four UPS sarcoma cell lines SHEF UPS01 (RRID:CVCL_C8V9), SHEF UPS02 (RRID:CVCL_C8VA), SHEF UPS03 (RRID:CVCL_C8VB) [21] and SHEF UPS04 (RRID:CVCL_C8VI) [22] were cultured as previously described and provided by Dr. Karen Sisley (University of Sheffield). The IEC‐56 cell line was generated and validated (see Section 2.4 and Fig. 2) as part of this study and does not have a Research Resource Identifier. All cells were routinely checked for mycoplasma contamination, and experiments were carried out with mycoplasma‐free cells. Cells were verified by short tandem repeat (STR) profiling (conducted in August 2022 for the IEC‐56 cell line and February 2023 for the SHEF UPS01‐04 cell lines by the Institute of Genetics and Cancer Technical Services).

### 2.6 End‐point drug efficacy studies

Cells were seeded onto flat‐bottom 384‐well plates (Greiner, Kremsmünster, Austria) followed by compound addition (see complete list and details in Table S1) 24‐h postseeding. After 72‐h treatment, cells were stained with 1 μm Hoechst 33342 (Thermo Scientific, Waltham, MA, USA) for 1 h and imaged using the ImageXpress Micro XL device (Molecular Devices, San Jose, CA, USA) with four fields of view (10× objective) to obtain the nuclei count in each well. The results were analysed using the metaxpress software (Molecular Devices). Results of drug combination studies were analysed using the synergyfinder+ software [23] and primarily the ZIP model [24].

3D spheroids were allowed to aggregate for 72‐h postseeding in ultra‐low attachment 384‐well plates (S‐BIO, Hudson, NH, USA) and subsequently treated with compounds for a further 72 h. For end‐point measurement, spheroids were incubated with the PrestoBlue Cell Viability Reagent (Invitrogen) for 3 h at 37 °C, and the fluorescence intensity was measured using the Spark 20 M plate reader (Tecan, Männedorf, Switzerland). IC_50_ values for the drugs tested in both 2D and 3D were calculated using Prism 10 software (GraphPad, San Diego, CA, USA).

The initial viability of tumour slices was obtained by incubating them immediately following slicing with PrestoBlue Cell Viability Reagent for 1 h at 37 °C. The media was transferred to a 96‐well flat‐bottom plate in triplicate, and the fluorescence intensity was measured using the Spark 20 M plate reader (Tecan). The slices were allowed to equilibrate for 24 h prior to drug treatment and were treated for 120 h with one media change at either 48 or 72 h following the start of treatment. The slices were subsequently incubated with PrestoBlue Cell Viability Reagent for 1 h at 37 °C, and fluorescence intensity was measured as above.

### 2.7 Genomic analysis

For the full description of the whole exome sequencing procedure, see the Methods S1.1–S1.3. The same genomic processing steps were carried out on both our in‐house samples as well as published raw (Fastq) UPS data generated in the TCGA Sarcoma project (*n* = 44) [10]. All sequenced data were aligned to the GRCh38 human reference genome using bwa‐0.7.17 [25], duplicates marked and base quality scores recalibrated with the Genome Analysis Toolkit (GATK) v4 [26] within the bcbio 1.0.6 pipeline (see Methods S1: Mapping of sequenced reads). Mutations were defined as genomic events encompassing both SNVs (single nucleotide variants) and INDELS (short insertions and deletions). Mutations were called using a majority vote system from three variant callers: VarDict [27], Mutect2 [28] and Freebayes [29] and were subsequently filtered to remove technical artefacts and then to remove common and likely nonfunctional variants through a score‐based model approach (Methods S1: Variant calling and classification). In brief, this involved following a previously published pipeline for cumulative scoring using well‐annotated cancer databases, population single nucleotide polymorphisms and predictive modelling approaches to classify variants as either benign, likely benign, variants of unknown significance, likely oncogenic or oncogenic [30]. To focus on translatable results, likely oncogenic and oncogenic mutations were combined into a single group defined as ‘high‐confidence oncogenic’ (HCO) mutations.

CN data were generated using CNVkit (V 0.9.3) using default thresholds to define a ‘gain’ (CN = 3), ‘amplification’ (CN > 3), ‘shallow deletion’ (CN = 1) or ‘deep deletion’ (CN = 0) [31] with (a) patient tumours and PDX models: matched normal blood‐derived genomic backbone datasets where available and where not, an average down sampled normal dataset generated from these normal samples; (b) cell lines: data from a nontumour cell line (HCC1143_BL) as a background normal dataset. All normal datasets were processed in parallel to the tumour samples through the same alignment and processing pipelines. Analysis of tumour mutational burden (TMB) was carried out on all mutations, whilst genes of interest for SNV analysis were focussed on those defined as likely pathogenic and pathogenic only. CN analysis was focussed on genes with related cancer functions as defined by cancer gene sets taken from gene lists defined by OncoKB [32].

Analysis of variants was performed using the R package maftools [33]. TCGA SNV data were used as a sense check of the Edinburgh solid tumours, cell lines and PDX models. Principal component analysis (PCA) was employed on a binary matrix of events for either HOC mutations or oncoKB cancer genes containing a CN event, using the prcomp function in R. Heatmaps for CN analysis were plotted using a custom R script to perform hierarchical clustering. Rows and columns with zero variance were filtered out, and clustering was conducted using Manhattan distance for rows and correlation distance for columns, both with Ward's method.

### 2.8 Reverse‐phase protein array

Cells were lysed in RIPA buffer supplemented with cOmplete™ EDTA‐free Protease Inhibitor Cocktail (Roche, Basel, Switzerland), sodium fluoride and sodium orthovanadate. Protein concentration was quantified using Pierce™ BCA Protein Assay Kits (Thermo Scientific) according to the manufacturer's instructions. Samples were diluted to 2 mg·mL^−1^and profiled on the Quanterix and Innopsys reverse‐phase protein array (RPPA) platforms by the Host and Tumour Profiling Unit (University of Edinburgh) as described previously [34]. Briefly, samples were denatured at 95 °C for 5 min and serially diluted from 1.5 mg·mL^−1^ to 0.1875 mg·mL^−1^. All four sample dilutions were spotted onto single pad ONCYTE® SuperNOVA nitrocellulose slides (Grace Bio‐Labs, Bend, OR, USA) with the 2470 Arrayer platform (Quanterix, Billerica, MA, USA). The 1× Reblot Plus Strong Solution (Millipore) was used for antigen retrieval followed by blocking in SuperBlock T20 (TBS) (Thermo Scientific). The slides were subsequently incubated with primary antibodies (Table S4) diluted 1 : 250 in SuperBlock T20, followed by incubation with Dylight‐800‐labelled anti‐species antibodies (New England BioLabs, Ipswich, MA, USA) diluted 1 : 2500 in SuperBlock T20. The arrays were imaged with the Innoscan 710 scanner (Innopsys, Carbonne, France) and the relative fluorescence intensity value corresponding to protein abundance was quantified using the Mapix software (Innopsys).

## 3 Results

### 3.1 Genomic landscape of UPS across patient samples, PDX models and cell lines

To characterise the genomic landscape of UPS and assess the relevance of laboratory models for *in vitro* assays, we performed whole exome sequencing (WES) on tumour‐normal tissue pairs from 20 patients, four established cell lines (SHEF UPS01, SHEF UPS02, SHEF UPS03 and SHEF UPS04 [21, 22]), and three novel PDX models (IEC‐16, IEC‐56 and SARC‐395). WES identified a median of 175 total mutations per sample (SNVs and INDELs) across the cohort (range: 125–9037) (Fig. S2A). PDX models exhibited significantly more total mutations (median: 4464, 2.0% HCO) than cell lines (median: 218.5, 7.3% HCO) or patient tumours (median: 159.5, 8.5% HCO), most likely due to the challenges involved in the disambiguation between human and murine reads in the PDX models as reflected by the lower percentage of reads defined as HCO (Fig. S2A,B). For comparison, UPS tumour samples from the TCGA Sarcoma Project (*n* = 44) had a median of 187.5 mutations (8.1% HCO) (Fig. S2).

**Fig. 1:**
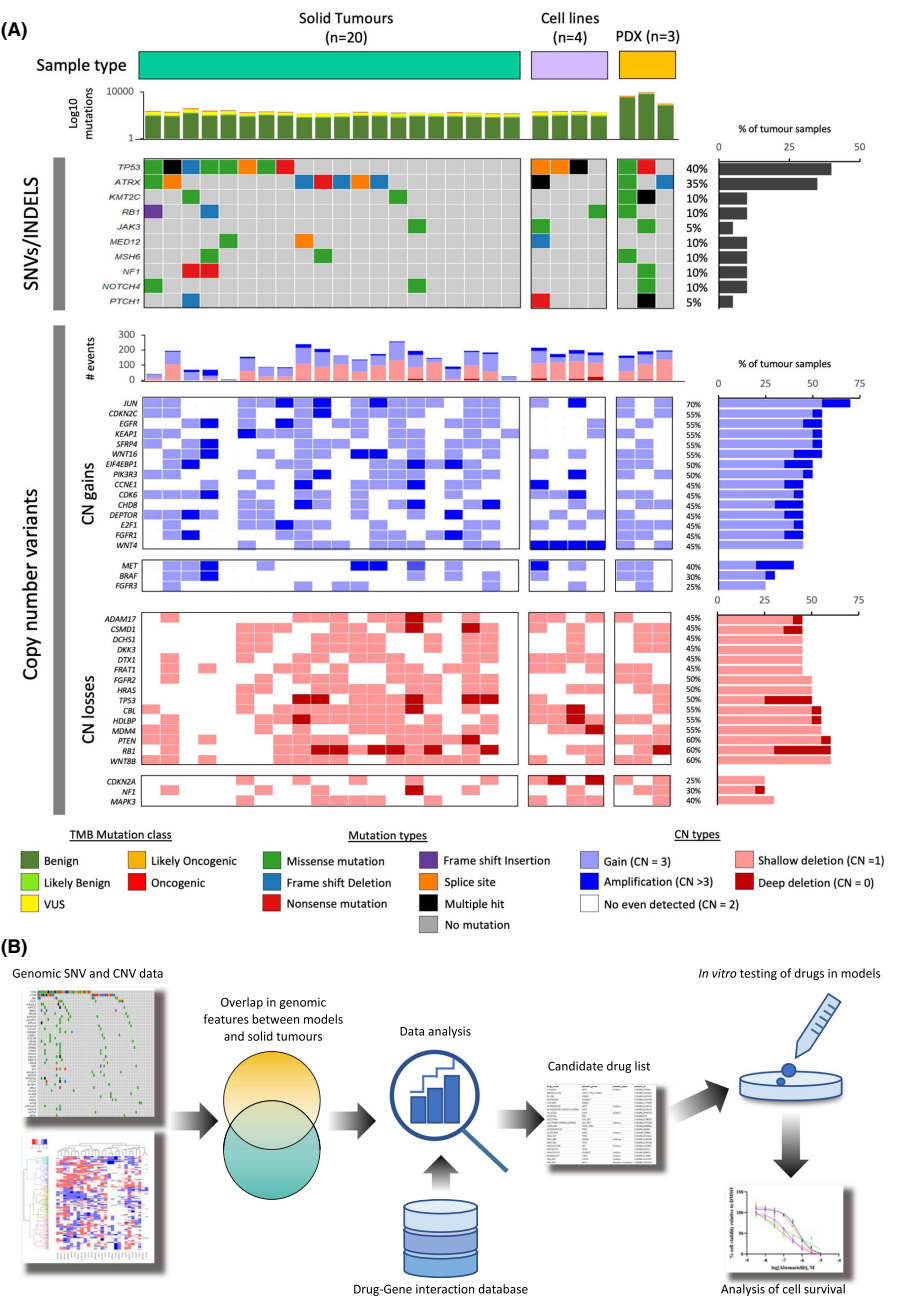
The combined genomic landscape of UPS tumours, cell lines and PDX models. (A) Whole exome genomic analysis of UPS samples from solid tumours (teal), cell lines (purple) and PDX mouse models (gold; top bars). SNVs/INDELS: All detected mutations are plotted along the top, split by mutation classification. Likely pathogenic and pathogenic mutations detected in more than one tumour sample and at least one model sample are shown on an oncoplot with the percentage of tumour events plotted to the right. Bar plot of the number of altered samples is shown on the right. Copy number variants: plot of total CN counts over cancer genes is plotted above. Frequent copy number events are plotted below (blue = CN gain, red = CN loss) with bar plots of the percentage of CN events in tumour samples shown to the right. Select (*n* = 3) genes of interest are also plotted for CN gain and loss. Colour codes for mutation class, mutation type and CN type are shown below. CN, copy number; SNV, Single nucleotide variant; TMB, Tumour mutational burden. (B) Schematic workflow for the interrogation of genomic data resulting in the identification of therapeutic agents for *in vitro* testing.

Focusing on HCO events, similar mutational events were seen across the patient‐derived tumour samples and the cell and PDX models, with PCA revealing no independent grouping of tumours based on sample source (Fig. S3). Although frequently mutated genes across the cohort were uncommon, these were seen in both the patient tumours and cell models including those in *TP53* (40% patient tumours, 75% cell lines, 67% PDX models), *ATRX* (35% patient tumours, 35% cell lines, 67% PDX models) and *RB1* (10% patient tumours, 25% cell lines, 0% PDX models) (Fig. 1A and Figs S3 and S4). Other mutations of note include *JAK3* (10% patient tumours, 25% cell lines, 33% PDX models) and *NF1* (10% patient tumours, 0% cell lines, 25% PDX models) (Fig. 1A). Comparison of oncogenic mutations to those in the TCGA data processed through our analysis pipeline revealed similar frequencies of mutations to those detected in both our solid tumour and model systems (Fig. S4).

Consistent with previous studies [10, 12], CN alterations were more prevalent than mutations in UPS, affecting a median of 153 well‐defined cancer genes in patient tumours and a median of 192 genes in both cell lines and PDX models. As observed with mutational data, PCA and hierarchical clustering of CN‐altered cancer genes did not reveal distinct sample‐specific groupings. Instead, CN patterns showed some similarity between solid tumours and model systems (Figs S3B and S5).

Across the cohort, we noted frequent perturbation of genes associated with several pathways including cell cycle regulation, receptor tyrosine kinase (RTK)‐RAS, NOTCH and WNT signalling. Frequent CN gains were observed in *JUN* (70% patient tumours, 50% cell lines, 66% PDX models), *EGFR* (55% patient tumours, 25% cell lines, 33% PDX models) and *CDK6* (45% patient tumours, 75% cell lines, 66% PDX models) with notable gains also seen in *MET* (40% patient tumours, 50% cell lines, 66% PDX models; Fig. 1A). CN losses were commonly detected in *WNT8B* (60% patient tumours, 50% cell lines, 66% PDX models), *RB1* (60% patient tumours, 50% cell lines, 66% PDX models) and *PTEN* (60% patient tumours, 0% cell lines, 100% PDX models) with notable losses also seen in *CDKN2A* (25% patient tumours, 75% cell lines, 66% PDX models; Fig. 1A).

### 3.2 Identification of actionable alterations

Based on the common genomic alterations present in the patient samples, PDX models and cell lines, we utilised the Drug‐Gene Interaction database (www.dgidb.org) to identify potentially actionable drug targets which were taken forward for testing (Fig. 1B and Table 1). Due to the low frequency of SNV events, compounds were selected to target primarily CN changes, including gains in *CDK6*, *MET*, *KIT*, *PIK3R1/3, KMT2A*, *NOTCH1/3* and *FGFR1/3*, and losses in *TP53* and *PTEN* as well as one SNV (*JAK3*). Interestingly, several drugs targeting the above alterations have previously been studied in STS. These include: cyclin‐dependent kinase (CDK)4/6 inhibitors abemaciclib [35] and palbociclib [36], vascular endothelial growth factor receptor (VEGFR) and KIT inhibitor anlotinib [37, 38], γ‐secretase inhibitor nirogacestat [39], poly (ADP‐ribose) polymerase (PARP) inhibitor olaparib [40] and MEK inhibitor trametinib [41]. The following drugs that had shown promising results in preclinical STS models were also selected: FGFR inhibitors erdafitinib and infigratinib, PARP inhibitor niraparib and pan‐phosphatidylinositol 3‐kinase (PI3K) inhibitor ZSTK474 [42, 43, 44]. This was complemented by drugs not previously studied in STS: (JAK)3‐specific inhibitor FM‐381 and a broad‐spectrum JAK inhibitor tofacitinib, the latter which has been approved for the treatment of rheumatoid arthritis [45]. The amplification of *KMT2A* could be targetable through inhibition of components of the COMPASS complex required for KMT2A's methyltransferase function, namely menin [46] with revumenib and WDR5 [47] with OICR‐9429. Taken together, a panel of 14 compounds was selected for a primary screen in UPS cell lines.

**Table 1:** Drugs chosen for a screen based on the whole exome sequencing analysis of UPS patient biopsies, cell lines and PDX models.

SNV/CNV	Source	Drug	Target
*CDK6* amplification	Cell lines, PDX models and patient tumours	Abemaciclib	CDK4/6 inhibitor
		Palbociclib	CDK4/6 inhibitor
*FGFR1* and *FGFR3* amplifications	Cell lines (*FGFR3* only), PDX models (both) and patient tumours (both)	Erdafitinib	FGFR inhibitor
		Infigratinib	FGFR inhibitor
*JAK3* mutation	Cell lines, PDX models and patient tumours	FM‐381	JAK3 inhibitor
		Tofacitinib	JAK inhibitor
*KIT* amplification	Cell lines, PDX models and patient tumours	Anlotinib	VEGFR inhibitor
*KMT2A* amplification	PDX models and patient tumours	OICR‐9429	WDR5 inhibitor
		Revumenib	Menin inhibitor
*MAP2K2, BRAF* and *MET* amplifications	Cell lines, PDX models and patient tumours	Trametinib	MEK inhibitor
*NOTCH1* and *NOTCH3* amplifications	Cell lines, PDX models and patient tumours	Nirogacestat	γ‐secretase inhibitor
*TP53* and *PTEN* loss	Cell lines (*TP53* only), PDX models (both) and patient tumours (both)	Olaparib	PARP inhibitor
		Niraparib	PARP inhibitor
*PIK3R2* and *PIK3R3* amplifications	Cell lines, PDX models and patient tumours	ZSTK474	PI3K inhibitor

### 3.3 Generation of a novel PDX‐derived IEC‐56 UPS cell line

There is a limited number of well‐annotated UPS cell lines available for drug screening [48, 49, 50, 51] and as UPS tumours are characterised by complex and variable genomic changes this highlights the need to use a range of cell lines with diverse genomic backgrounds for drug testing. We were able to derive a novel UPS cell line from the IEC‐56 PDX model to complement the four UPS cell lines (SHEF UPS01‐UPS04) in the drug screening experiments.

**Fig. 2:**
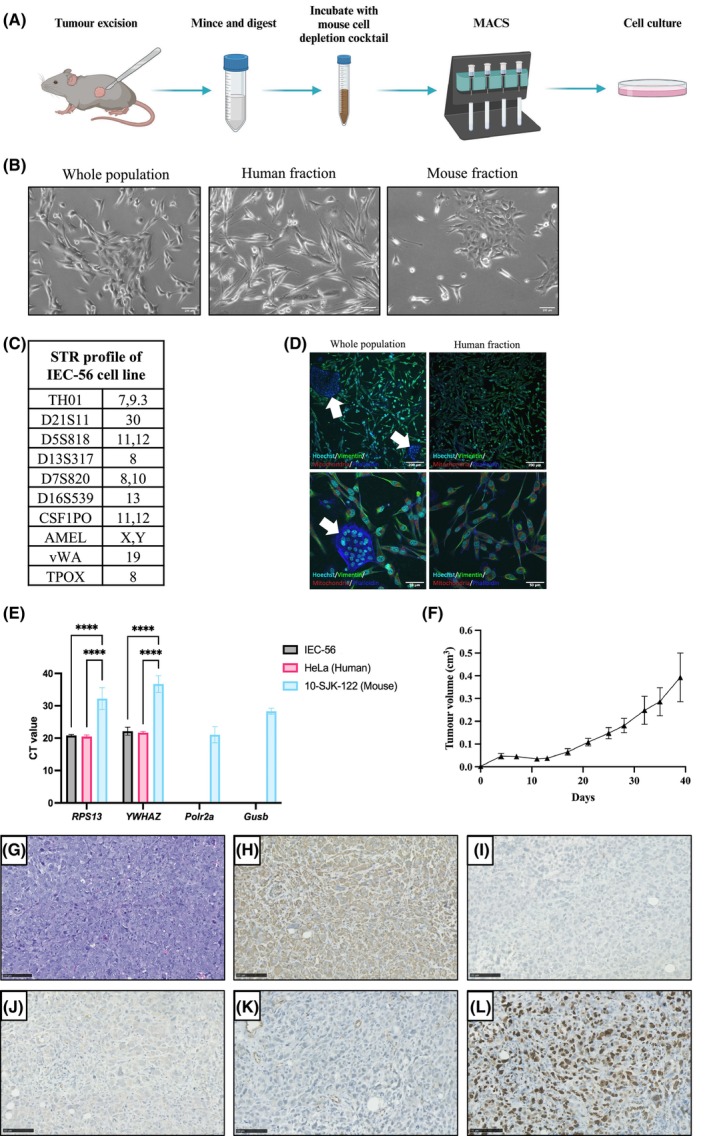
Characterisation of the novel PDX‐derived IEC‐56 cell line. (A) Schematic diagram of the IEC‐56 cell line derivation process. (B) Representative brightfield images of cells from the whole population (left), human fraction (middle) and mouse fraction (right) obtained from the dissociated and mouse‐depleted IEC‐56 tumour (*n* = 1). Scale bars = 100 μm. (C) STR profile of the IEC‐56 UPS cell line. (D) Representative images of immunofluorescent staining of cells from the whole population (left) and human fraction (right) obtained from the dissociated and mouse‐depleted IEC‐56 tumour for human mitochondria, human and mouse vimentin and actin (phalloidin) (*n* = 3). Images were taken 10× (top row) and 40× (bottom row) magnification. The white arrow points to a cluster of mouse cells characterised by the absence of vimentin and human mitochondria staining. Scale bars = 200 μm (top row) and 50 μm (bottom row). (E) qRT‐PCR validation of the human IEC‐56 cell line purity. Primers targeting two widely used human (*RPS13* and *YWHAZ*) and mouse (*Polr2a* and *Gusb*) reference genes were used. The error bars represent the standard deviation from the mean (*n* = 3). The results were analysed using a two‐way ANOVA with Tukey's multiple comparisons test (****P < 0.0001). (F) Growth of the IEC‐56 cell line in mice following subcutaneous injection. The error bars represent the standard deviation from the mean tumour volume at each time point (*n* = 10 from five mice). (G–M) Representative H&E (G) and immunohistochemical staining of the tumours formed by subcutaneous IEC‐56 cell line injections for vimentin (H), cytokeratin (I), smooth muscle actin (J), CD31 (K) and Ki67 (L) (all *n* = 3). Scale bars = 100 μm.

The human cell population was obtained by mouse cell‐depleting freshly dissociated tumour cells with magnetic‐activated cell sorting (Fig. 2A,B). The IEC‐56 cell line was STR profiled (Fig. 2C), and the absence of mouse cells in the cell line population was confirmed by immunofluorescence (Fig. 2D). The purity of the IEC‐56 cell line was further validated by RT‐PCR using human‐ and mouse‐specific reference gene primers (Fig. 2E). The IEC‐56 cells were tumouMrigenic when injected into immunocompromised mice (Fig. 2F) and the representative H&E stain of the resulting tumours is shown in Fig. 2G. The tumours were vimentin‐positive (Fig. 2H) and negative for cytokeratin (Fig. 2I) and smooth muscle actin (Fig. 2J). The tumours showed signs of developing vasculature (Fig. 2K) and contained a high proportion of proliferating cells as demonstrated by Ki67‐positive staining (Fig. 2L).

### 3.4 CDK4/6, FGFR, MEK and PI3K inhibitors show the highest efficacy in UPS cell lines

**Fig. 3:**
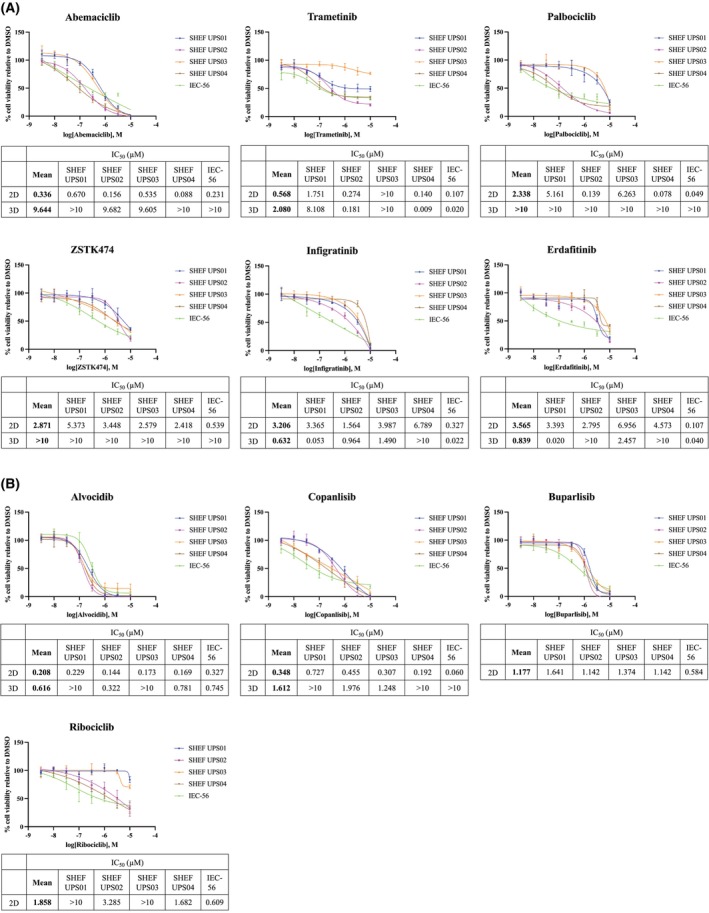
CDK4/6, FGFR, MEK and PI3K inhibitors show the highest efficacy in UPS cell lines. (A) Log‐dose response curves of compounds from the primary screen to which at least one UPS cell line was sensitive in 2D (top two rows; the error bars represent the standard deviation from the mean of *n* = 3 biological replicates). The tables underneath summarise the mean IC_50_ values of the cell lines for which exact IC_50_ values could be determined and the individual IC_50_ values for each cell line in 2D and 3D culture. (B) Log‐dose response curves of efficacious compounds identified in the secondary screen tested in the UPS cell lines grown in 2D cultures (the error bars represent the standard deviation from the mean of *n* = 3 biological replicates). The tables underneath summarise the mean IC_50_ values of the cell lines for which exact IC_50_ values could be determined and the individual IC_50_ values for each cell line. Buparlisib and ribociclib were not tested in 3D cultures.

The full results of the primary screen conducted on the five UPS cell lines are summarised in Tables S5 and S6 The CDK4/6 inhibitor abemaciclib was the most efficacious drug tested in 2D as measured by nuclei counts with an average IC_50_ of 0.335 μM (range: 0.088–0.670 μm) (Fig. 3A and Table S5). Palbociclib, another CDK4/6 inhibitor, performed similarly to abemaciclib in SHEF UPS02, SHEF UPS04 and IEC‐56 cell lines, but had considerably higher IC_50_ values in SHEF UPS01 (5.161 μm) and SHEF UPS03 (6.263 μM). Trametinib had the second lowest average IC_50_ value in the screen (0.568 μm; range: 0.107–1.751 μm) although the SHEF UPS03 cell line was resistant to trametinib (IC_50_ value above 10 μm) (Fig. 3A and Table S5). Sensitivity, defined as an IC_50_ value below 1 μm, to ZSTK474, infigratinib and erdafitinib was seen in at least one of the lines (Fig. 3A and Table S5).

In 3D spheroid cultures, infigratinib and erdafitinib were the most efficacious compounds tested as measured by the PrestoBlue reagent reduction, with average IC_50_ values of 0.632 and 0.839 μm, respectively (range: 0.022–1.490 μm and 0.020–2.457 μm, respectively) (Fig. S6 and Table S6). In the majority of the UPS cell lines, both drugs were more potent in 3D spheroids than in 2D cultures (Fig. 3 and Tables S5 and S6). In line with 2D results, SHEF UPS03 was resistant to trametinib while SHEF UPS02, SHEF UPS04 and IEC‐56 had lower 3D IC_50_ values (0.181, 0.009 and 0.020 μm, respectively) compared with 2D cultures. Abemaciclib performed poorly in the 3D screen with IC_50_ values just below 10 μm in SHEF UPS02 and SHEF UPS03 cell lines with the other cell lines having IC_50_ values > 10 μm.

Based on the above results, additional CDK4/6, MEK and PI3K inhibitors were chosen for further assessment in a secondary screen in 2D (Table S7). Multiple genomic changes in the PI3K/AKT pathway were identified in UPS samples (Fig. 1) providing the rationale behind testing further PI3K inhibitors despite ZSTK474's lack of efficacy in the majority of the cell lines (Tables S5 and S6). Additionally, inhibitors specific to one or more of the four PI3K subunits were included to assess whether targeting different PI3K subunits is efficacious in the UPS cell lines. The nonspecific CDK inhibitor alvocidib and pan‐PI3K inhibitor copanlisib were the two compounds identified in the secondary screen to which the UPS cell lines were sensitive (Fig. 3B). Alvocidib had the lowest average IC_50_ value of all drugs tested in 2D (0.208 μm, range: 0.144–0.327 μm) and was more efficacious than abemaciclib in 3D cultures. Similarly, copanlisib outperformed the pan‐PI3K inhibitor ZSTK474 tested in the primary screen in both 2D and 3D, although only SHEF UPS02 and SHEF UPS03 had IC_50_ values below 10 μm in spheroid cultures. The PI3K subunit‐specific inhibitors performed poorly in the secondary screen and none of the MEK inhibitors showed similar efficacy to trametinib (Tables S8). Table S9 provides a summary of the sensitivity profiles of each cell line together with the associated genomic alterations.

### 3.5 Combination of trametinib and infigratinib is synergistic in the majority of UPS cell lines tested

**Fig. 4:**
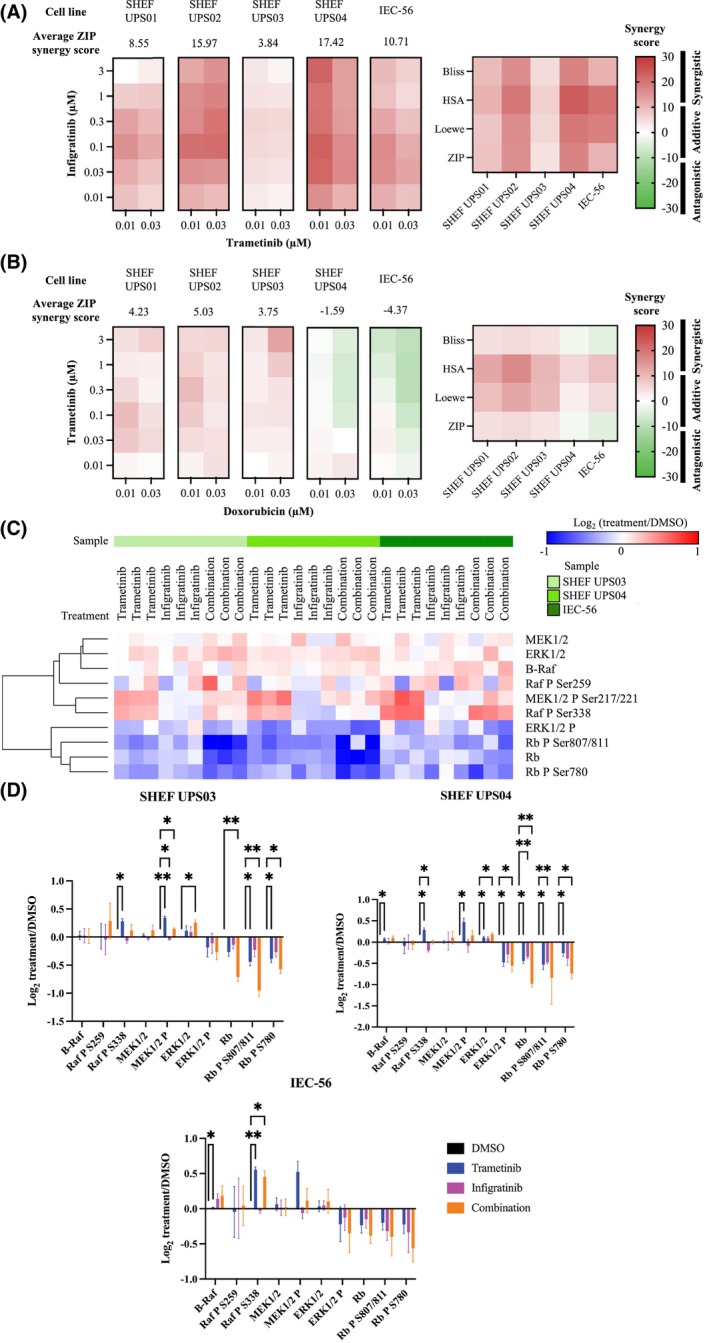
Combination of trametinib and infigratinib is synergistic in UPS cell lines. (A) Heat maps summarising the ZIP synergy scores (left) and all four synergy scores (right) of selected infigratinib and trametinib doses in the UPS cell lines grown in 2D (*n* = 3 biological replicates). The average ZIP synergy score for the selected dose matrix is shown above each heat map. (B) Heat maps summarising the ZIP synergy scores (left) and all four synergy scores (right) of selected doxorubicin and trametinib doses in the UPS cell lines grown in 2D (*n* = 3 biological replicates). The average ZIP synergy score for the selected dose matrix is shown above each heat map. (C) Summary of changes in levels of selected proteins from the RPPA assay following a 24‐h treatment period (*n* = 3 biological replicates). The protein level changes were hierarchically clustered using Euclidean distance. (D) Graphical representation of the results shown in (C). The error bars represent the standard deviation from the mean (*n* = 3 biological replicates). The results were analysed using a two‐way ANOVA with Dunnett's multiple comparisons test (*P < 0.05; **P < 0.01).

Abemaciclib, copanlisib and trametinib were further tested in combinations with each other to assess whether they would exhibit additive or synergistic effects. Abemaciclib was selected over alvocidib based on its previous use in STS clinical trials. Trametinib was also combined with infigratinib based on recent evidence of their synergy in models of cancers of unknown primary [52]. Out of the drug combinations tested, only infigratinib paired with low doses of trametinib (0.01 and 0.03 μm) showed synergy, defined as a synergy score above 10 [53], across most UPS cell lines (Fig. 4A) with the highest average ZIP scores and maximum ZIP scores (ZIP_max_) seen for SHEF UPS02 (15.97; ZIP_max_ = 21.07) and SHEF UPS04 (17.42; ZIP_max_ = 22.83). SHEF UPS03 had the lowest average score out of the five UPS cell lines (3.84; ZIP_max_ = 5.59), indicating that infigratinib was not able to overcome the resistance of SHEF UPS03 to trametinib. The synergy of infigratinib and trametinib at low doses of trametinib identified by the ZIP model in SHEF UPS02, SHEF UPS04 and IEC‐56 cell lines was consistent across the other three synergy models (Fig. 4A). The HSA model also indicated synergy (average score of 10.99) in the SHEF UPS01 cell line; however, this result was not supported by the other three models. The synergy scores for the whole dose matrix of the infigratinib and trametinib combination are shown in Table S10.

We then looked at possible synergy between trametinib and UPS standard‐of‐care doxorubicin. No synergy was identified using the ZIP model with only 3 μm trametinib combined with 0.03 μm doxorubicin in the SHEF UPS03 cell line showing a synergy score above 10 (Fig. 4B). The average ZIP synergy scores for the selected dose matrix shown in Fig. 4B ranged from −4.37 (IEC‐56) to 5.03 (SHEF UPS02). HSA and Loewe models indicate that doxorubicin and trametinib are synergistic in SHEF UPS02 (average scores of 16.75 and 12.4, respectively) with the latter model also pointing to synergy in SHEF UPS01 (12.49) and SHEF UPS03 (10.1). Due to the lack of consistency across the models and low synergy scores, we decided to look further at the observed synergy between trametinib and infigratinib rather than the combination with doxorubicin.

To identify the effects of trametinib and infigratinib on different signalling pathways in the cell lines, we performed a RPPA experiment (Fig. 4C and full results in Fig. S7). SHEF UPS03, SHEF UPS04 and IEC‐56 cell lines were treated with 10 nM trametinib and 100 nM infigratinib, either as monotherapies or in combination. These doses were selected as their combination gave the highest ZIP synergy score across the five UPS cell lines (Fig. 4A). Trametinib treatment led to downregulation of pERK in all three cell lines, although it was only significant in the SHEF UPS04 cell line. Combined treatment with infigratinib did not result in a further reduction in pERK. The reduction in pERK was accompanied by an upregulation of pMEK as has been described previously following trametinib treatment [54, 55]. However, this increase was not as marked when trametinib was combined with infigratinib. In addition, increased phosphorylation of Raf (S338) was seen in trametinib‐treated cells which was reduced in the combination‐treated cells. As expected, MEK levels were unchanged in all cell lines following the treatments and ERK was modestly upregulated by the combination treatment in SHEF UPS03 and by both trametinib and combination treatments in SHEF UPS04.

All three treatments resulted in lower RB levels in the SHEF UPS04 cell line with the trametinib and combination treatment also leading to a reduction in pRB levels (Fig. 4D). Combination treatment resulted in significantly lower RB and pRB (S780) levels compared to trametinib (*P* = 0.0002 and *P* = 0.03, respectively) and infigratinib (*P* = 0.03 and *P* = 0.01, respectively), indicating that some of the synergy seen with the combination treatment could be due to enhanced cell cycle arrest. This was also observed in the SHEF UPS03 cell line with combination treatment reducing RB and pRB (S807, S811) levels significantly more than both trametinib (*P* = 0.006 and *P* = 0.04, respectively) and infigratinib (*P* = 0.01 and *P* = 0.02, respectively). Trametinib and combination treatments reduced pRB levels at all sites in SHEF UPS03 cells with the combination also lowering levels of RB. This suggests that the inherent resistance to trametinib of SHEF UPS03 cells negates the effects of the treatment on RB and its phosphorylation. In the IEC‐56 cell line, the changes in RB and pRB levels followed the same pattern as the other two cell lines but these were found to not be significant, likely driven by higher variability in response to treatment seen in this cell line (Fig. 4D). Analysis of other protein level changes in the RPPA assay did not reveal any consistent changes following single agent or combination treatments (Fig. S7).

### 3.6 UPS tumour slices as a viable model for validation of drug efficacy

**Fig. 5:**
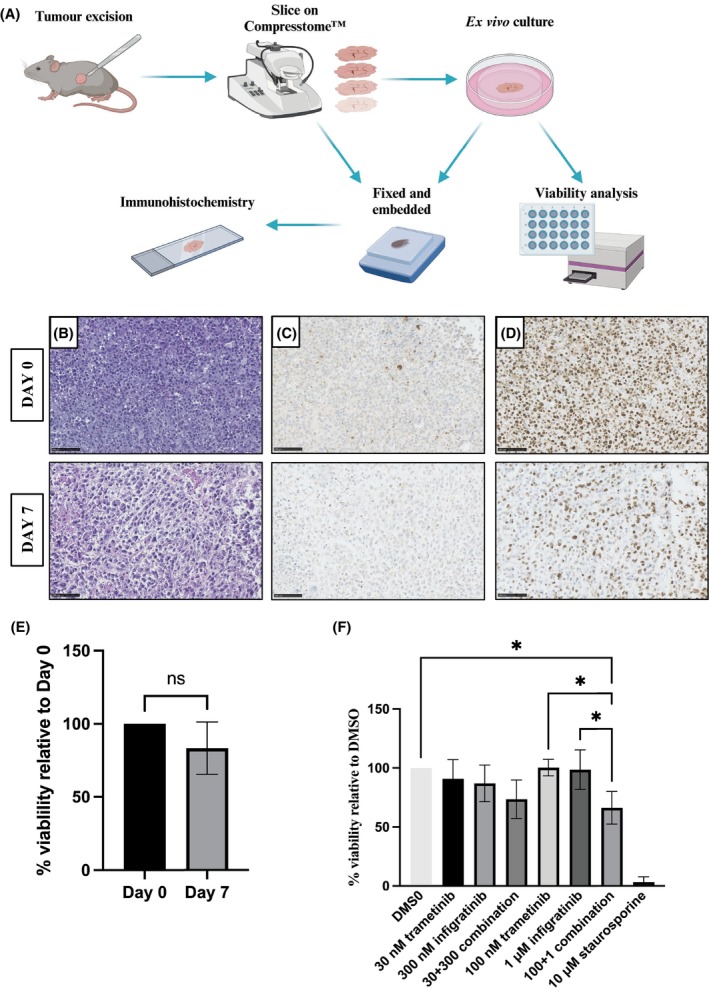
Use of *ex vivo* UPS PDX tumour slice cultures for validation of drug efficacy. (A) Outline of the tumour slicing process and downstream analysis. (B–D) Representative H&E (B) and immunohistochemical staining of the IEC‐56 PDX tumour slices for cleaved caspase‐3 (C) and Ki67 (D) at two time points postslicing. Scale bars = 100 μm. (E) Viability of IEC‐56 PDX tumour slices relative to the start of culture. The results were analysed using a two‐tailed *t*‐test (ns = not significant). The error bars represent the standard deviation from the mean (*n* = 4 biological replicates from 4 different tumours). (F) The effect of treatment with trametinib, infigratinib and their combination on tissue slice viability. Staurosporine treatment (10 μm) was used as a positive control. The error bars represent the standard deviation from the mean (*n* = 4 biological replicates). The results were analysed using a one‐way ANOVA with Tukey's multiple comparisons test (*P < 0.05).

To bridge the gap between *in vitro* and *in vivo* drug testing in UPS, a protocol for *ex vivo* tumour slice cultures derived from the IEC‐56 PDX model was established (Fig. 5A). Over a seven‐day culture period, the slices maintain their morphology (Fig. 5B) although they become less compact as evidenced by larger intercellular spaces. The slices do not exhibit an increase in apoptosis (Fig. 5C) and remain proliferative (Fig. 5D), albeit the proportion of proliferating cells is reduced on day 7 compared to the freshly sliced tumour. The viability of the slices is not significantly changed over 1 week of culture (Fig. 5E). To test whether the synergy between trametinib and infigratinib is maintained in a more complex, *ex vivo* culture system the slices were treated with two doses of trametinib, infigratinib and their combinations (Fig. 5F). Treatment with 10 μm staurosporine was used as a positive control and demonstrated that a near complete loss of viability to 3.2% relative to the DMSO control (range: 0.0–9.8%). The combination of 100 nM trametinib and 1 μ infigratinib was the only treatment to significantly reduce slice viability (mean viability of 66.2%) relative to DMSO control. Following the treatment with this combination, the slice viability was also significantly lower compared to both single compound treatments. The higher doses of both drugs required to observe efficacy in the slices compared to 2D cultures likely reflect the more complex tissue architecture of the tumour slice model and drug concentration gradients across the tissue piece. This result further demonstrates the increased efficacy of trametinib when combined with infigratinib in preclinical UPS models.

## 4 Discussion

In this study, we set out to drive rational selection and testing of drug compounds in both cell line and PDX‐derived models, based on genomic analysis of a local UPS patient tumour cohort. To do so, we employed a comprehensive analysis of the genomic landscape in 4 cell lines and 3 PDX models and related these to the findings of analysis of 20 UPS patients with applied oncogenic filtering methods to assess both SNV and CN events that are potentially therapeutically targetable.

As with previous studies, our UPS tumour samples, cell lines and PDX models were dominated by complex genomic perturbations such as CN changes, rather than SNV events [10, 14]. That being said, a small number of genes did demonstrate frequent SNV events. The most common of these were *TP53* and *ATRX* tumour suppressor genes. *TP53* has been widely reported to be the most prevalent genomic alteration in UPS [10, 12, 13, 14, 15, 16]. *TP53* was also frequently altered through CN loss events in the tumour samples, usually in tumours without a *TP53* SNV. Similar mutation and CN events were also seen across the cell lines and PDX models. Our patient cohort also had a similar prevalence of *ATRX* mutations to a number of previous UPS studies including those reported by Zheng and colleagues (*n* = 16, 31% of cases) [16] as well as those observed in UPS samples in large studies such as the analysis of the MSK‐IMPACT data (*n* = 145, 21% of cases [14]) and the TCGA Adult Soft Tissue Sarcoma study (*n* = 44, 29.5% of cases [10]).

Previous studies have also reported genomic alterations in *RB1* in UPS [10, 14]. In our patient tumours, we note that 10% of cases had a mutation and 60% had a CN change, with similar events also seen in the cell lines and PDX models. We also report a comparable frequency of loss of the *CDKN2A* gene (25% in our tumour group) to those described by Bui and colleagues by analysing the Foundation One Medicine database (*n* = 372, 21% of cases) [15] and to that reported in the TCGA Adult Soft Tissue Sarcoma study (*n* = 44, 20.5% of cases) [10]. Other previously reported mutated genes in UPS such as *NF1*, *PTEN* and *KMT2C* [14, 16] were also found to be mutated in our samples.

Frequent CN alterations were found in genes associated with several pathways including cell cycle, RTK‐RAS, NOTCH and WNT signalling. We observe frequent copy number gains in *JUN*, *EGFR* and *CDK6*. Conversely, CN losses were commonly detected in *WNT8B*, *RB1* and *PTEN*. These alterations align with findings from TCGA. Collectively, these findings reinforce the notion that UPS exhibits complex widespread genomic instability, with alterations in key oncogenic and tumour suppressor pathways. Notably, our model systems appear to recapitulate this genomic complexity, making them valuable tools for further research and therapeutic development.

CDK4/6 inhibitors are routinely used and well tolerated in hormone receptor‐positive, HER2‐negative advanced breast cancer [56] and have been evaluated in a number of clinical trials recruiting patients with STS. These clinical studies have mainly focussed on liposarcoma patients due to *CDK4* amplification which is found in nearly all cases [57, 58]. However, recently Martin‐Broto *et al* reported activity in other STS subtypes and observed an above median progression‐free survival (PFS) in a single UPS case treated with palbociclib as part of a phase II trial which recruited patients overexpressing *CDK4* and without overexpression of *CDKN2A* [36]. Here, we saw activity with a number of CDK4/6 inhibitors across the cell lines with abemaciclib showing superior activity to palbociclib and ribociclib, consistent with the greater potency of abemaciclib [59]. Sensitivity to abemaciclib across the cell lines was associated with different genomic alterations including *CDK4* and *RB1* amplification and loss of *CDKN2A* (Table S9). A study of 560 cancer cell lines showed that overall, *TP53* mutations were associated with relative insensitivity to abemaciclib but that several highly sensitive cell lines did have *TP53* mutations [60]. Interestingly, *TP53* mutations did not confer resistance in the SHEF UPS03 and IEC‐56 cell lines and highlight the need to consider multiple genomic alterations as these lines had alterations in other related genes including loss of *CDKN2A* and pathogenic mutations in *RB1*.

The secondary screen identified the nonspecific CDK inhibitor alvocidib as the most efficacious drug across the UPS cell lines tested. Interestingly, a recent study by Mendiola and colleagues identified alvocidib as one of the most efficacious compounds in a drug screen performed on two angiosarcoma cell lines [61]. Alvocidib has also been demonstrated to reduce proliferation and metastatic potential of osteosarcoma cells [62]. We found alvocidib to be efficacious in 3D cultures of SHEF UPS02, SHEF UPS04 and IEC‐56 cell lines, in stark contrast to abemaciclib and palbociclib which showed no activity in 3D spheroids. This could be due to additional inhibition of CDK2 by alvocidib, as activation of the CDK2‐cyclin E pathway has been proposed as one of the resistance mechanisms to CDK4/6 inhibition [63] and targeting CDK2 in addition to CDK4/6 overcame resistance to CDK4/6 inhibitors in breast cancer [64, 65].

While the PI3K inhibitor ZSTK474 was only efficacious in the IEC‐56 cell line, all five UPS cell line 2D cultures were sensitive to the PI3K inhibitor copanlisib included in the secondary screen. To our best knowledge, copanlisib has not been previously studied in UPS while in the broader context of sarcoma it showed a lack of *in vivo* activity in osteosarcoma models [66] and efficacy in both *in vitro* patient‐derived synovial sarcoma cells [67] and *in vivo* gastrointestinal stromal tumour models [68]. It hence represents an exciting potential candidate for further study in UPS, either as monotherapy or in combination with other agents.

MEK inhibitors are now widely used in combination with RAF inhibitors for patients with RAS or RAF mutations most notably in melanoma and nonsmall cell lung cancer [69]. Here we found 3/5 UPS cell lines were sensitive to trametinib in the absence of activating mutations in the RAS/RAF/MEK pathway. Although *RAF1* amplifications and deletions were detected in the cell lines these did not correlate with sensitivity across the lines. Trametinib was found to be efficacious in only a small minority of sarcoma cell lines tested by Teicher *et al*. some of which had activating RAS mutations [70]. Similar results were also obtained in a screen with a broad panel of patient‐derived sarcoma organoids [71] indicating that sensitivity to trametinib is rare in sarcomas. This is supported by a Phase II trial of selumetinib in STS patients which showed no difference in progression‐free survival—although the mutational status of RAS and RAF was not determined [72].

Resistance to MEK inhibitors is a major clinical challenge and can develop due to feedback activation of additional signalling pathways resulting in reactivation of ERK activity [69]. For this reason, clinical trials have investigated the combination of MEK inhibitors with other agents in a range of tumour types including one study in STS where trametinib was combined with the multi‐kinase inhibitor pazopanib which targets VEGFRs. The combination did not improve PFS although the authours suggested that trametinib should be further investigated in STS patients with an overactive MAPK pathway [41]. Although activation mutations in the pathway in UPS are very rare one study has identified high expression of pERK in around 30% of UPS samples with expression being an independent predictor of survival [73]. Understanding the pathways that lead to activation of the MAPK pathway in UPS will be important to define new combination therapies and here we have shown that combining trametinib with the FGFR inhibitor infigratinib was synergistic. Our genomic analysis showed copy number disruption of *FGFR1/2* across the cell lines; a result reflected in our human UPS tumour datasets where *FGFR1* was CN altered in 60% of cases and *FGFR2* in 50%. Indeed, both infigratinib and erdafitinib were shown to be efficacious in UPS cell lines derived from a subset of patients carrying *FGFR2* amplification, while erdafitinib also reduced *in vivo* growth of tumours formed by one of the *FGFR2* amplified cell lines [42]. Cavazzoni and colleagues also reported in cancers of unknown primary with *FGFR2* amplification that the combination of infigratinib and trametinib was synergistic and reduced the activity of both the AKT/mTOR/p70S6K and the MAPK pathway beyond the effects of the monotherapies [52]. Our results indicate that in UPS part of the synergistic effects of the combination could be due to the ability of infigratinib to prevent the feedback activation of RAF/MEK following treatment with trametinib resulting in arresting cell cycle progression.

We also demonstrate the feasibility of using *ex vivo* tumour slices to validate *in vitro* drug screening results. While tumour slices have been successfully used for drug studies before [74], this is the first reported use of UPS tumour slices for drug screening purposes. Tumour slices bridge the gap between more expensive, low throughput and time‐consuming *in vivo* experiments and *in vitro* models that do not recapitulate the tissue architecture and related complexities of the tumour. We demonstrated that UPS tumour slices can be kept in culture for 7 days without a loss in viability, in line with previous findings [75]. A potential drawback of the UPS tumour slices is the decreasing cell proliferation rate within the tissue over time, which could underestimate the efficacy of compounds targeting the cell cycle, such as CDK4/6 inhibitors. Nevertheless, our results indicate that trametinib and infigratinib in combination should be investigated further in UPS and tumour slices can be a valuable model for compound validation in UPS prior to *in vivo* experiments.

## 5 Conclusions

In conclusion, we demonstrate that our *in vitro* models recapitulate the genomic changes seen in UPS patients. Four out of 10 drug classes included in the genomic data‐informed primary screen contained compounds with efficacy in multiple UPS cell lines tested including CDK inhibitors and the PI3K inhibitor copanlisib. We identified a promising, synergistic combination of the MEK inhibitor trametinib and FGFR inhibitor infigratinib that warrants further testing. The above results highlight the utility of our genomics‐led approach to identify efficacious drugs in rare and understudied tumour types without clear genetic drivers.

## Conflict of interest

The authors declare no conflict of interest.

## Author contributions

VGB, NOC, and PJM contributed to conceptualisation; VGB, PJM, MRD, ML, AA, and JPT contributed to methodology; PJM, MRD, JPT, and JCD contributed to analysis; PJM, MRD, MM, and AJO contributed to investigation; ATA, JD‐M, DSM, JM‐B, GG, DMS, LH, TRH, KS, and PHH contributed to resources; PJM, MRD, and JPT contributed to data curation; PJM, VGB, and JPT contributed to writing original draft; VGB, PJM, MRD, and AJO contributed to review and editing; VGB and PJM contributed to supervision; VGB contributed to project administration; VGB contributed to funding acquisition. All authors have read and approved the manuscript.

## Supporting information


**Fig. S1.** The patient‐derived xenograft tumours maintain the morphology and identity of the original patient tumours.
**Fig. S2.** The distribution of mutation types across the UPS Edinburgh patient tumours, TCGA patient tumours, cell lines and PDX models.
**Fig. S3.** The single nucleotide variant and copy number events in UPS Edinburgh patient tumours, cell lines and PDX models do not cluster together.
**Fig. S4.** The prevalence of most frequently mutated genes across the UPS Edinburgh patient tumours, TCGA patient tumours, cell lines and PDX models.
**Fig. S5.** Heatmap of copy number changes detected in the UPS Edinburgh patient tumours, cell lines and PDX models.
**Fig. S6.** Erdafitinib, infigratinib and trametinib were the most efficacious compounds tested in 3D UPS cultures.
**Fig. S7.** Full results of the RPPA analysis.
**Table S1.** Details of compounds used for drug screening.
**Table S2.** Details of qRT‐PCR primers.
**Table S3.** Details of antibodies used for IHC and IF.
**Table S4.** Details of antibodies used for RPPA.
**Table S5.** The IC_50_ values for all compounds tested in the primary drug screen on 2D cultures of all UPS cell lines.
**Table S6.** The IC_50_ values for all compounds tested in the primary drug screen on 3D cultures of all UPS cell lines.
**Table S7.** The list of drugs and their targets tested in the secondary screen.
**Table S8.** The IC_50_ values for all compounds tested in the secondary drug screen on 2D cultures of all UPS cell lines.
**Table S9.** The sensitivity profiles of the five UPS cell lines to selected drugs and the associated genomic alterations identified in them.
**Table S10.** The complete results of the trametinib and infigratinib or doxorubicin combinations screen on 2D cultures of UPS cell lines.
**Methods S1.** Supplementary methods.

## Data Availability

The genomic data generated for patient tumours, cell lines and PDX models presented in this manuscript are currently under upload to the European Genome‐Phenome Archive (EGA: https://ega‐archive.org/) and will be available upon reasonable request. The WGS reads from UPS samples sequenced in the TCGA Sarcoma project were downloaded in aligned BAM format from the National Cancer Institute Protected Data Cloud Portal (https://portal.gdc.cancer.gov/).
