# Supplementary material for: Genomics‐led approach to drug testing in models of undifferentiated pleomorphic sarcoma
Source: Mol Oncol. 2025 May 26;19(11):3223–42. doi: 10.1002/1878-0261.70059 (PMC12591313; doi:10.1002/1878-0261.70059)
Supplement: Supplementary file 1 — Fig. S1. The patient‐derived xenograft tumours maintain the morphology and identity of the original patient tumours. Fig. S2. The distribution of mutation types across the UPS Edinburgh patient tumours, TCGA patient tumours, cell lines and PDX models. Fig. S3. The single nucleotide variant and copy number events in UPS Edinburgh patient tumours, cell lines and PDX models do not cluster together. Fig. S4. The prevalence of most frequently mutated genes across the UPS Edinburgh patient tumours, TCGA patient tumours, cell lines and PDX models. Fig. S5. Heatmap of copy number changes detected in the UPS Edinburgh patient tumours, cell lines and PDX models. Fig. S6. Erdafitinib, infigratinib and trametinib were the most efficacious compounds tested in 3D UPS cultures. Fig. S7. Full results of the RPPA analysis. Table S1. Details of compounds used for drug screening. Table S2. Details of qRT‐PCR primers. Table S3. Details of antibodies used for IHC and IF. Table S4. Details of antibodies used for RPPA. Table S5. The IC50 values for all compounds tested in the primary drug screen on 2D cultures of all UPS cell lines. Table S6. The IC50 values for all compounds tested in the primary drug screen on 3D cultures of all UPS cell lines. Table S7. The list of drugs and their targets tested in the secondary screen. Table S8. The IC50 values for all compounds tested in the secondary drug screen on 2D cultures of all UPS cell lines. Table S9. The sensitivity profiles of the five UPS cell lines to selected drugs and the associated genomic alterations identified in them. Table S10. The complete results of the trametinib and infigratinib or doxorubicin combinations screen on 2D cultures of UPS cell lines. Methods S1. Supplementary methods. [file MOL2-19-3223-s001.docx]

**Fig.** **S1.** The patient-derived xenograft tumours maintain the morphology and identity of the original patient tumours.
**Fig.** **S2.** The distribution of mutation types across the UPS Edinburgh patient tumours, TCGA patient tumours, cell lines and PDX models.
**Fig.** **S3.** The single nucleotide variant and copy number events in UPS Edinburgh patient tumours, cell lines and PDX models do not cluster together.
**Fig.** **S4.** The prevalence of most frequently mutated genes across the UPS Edinburgh patient tumours, TCGA patient tumours, cell lines and PDX models.
**Fig.** **S5.** Heatmap of copy number changes detected in the UPS Edinburgh patient tumours, cell lines and PDX models.
**Fig.** **S6.** Erdafitinib, infigratinib and trametinib were the most efficacious compounds tested in 3D UPS cultures.
**Fig.** **S7.** Full results of the RPPA analysis.

**Table** **S1.** Details of compounds used for drug screening.

**Table** **S2.** Details of qRT-PCR primers.

**Table** **S3.** Details of antibodies used for IHC and IF.

**Table** **S4.** Details of antibodies used for RPPA.

**Table** **S5.** The IC50 values for all compounds tested in the primary drug screen on 2D cultures of all UPS cell lines.

**Table** **S6.** The IC50 values for all compounds tested in the primary drug screen on 3D cultures of all UPS cell lines.

**Table** **S7.** The list of drugs and their targets tested in the secondary screen.

**Table** **S8.** The IC50 values for all compounds tested in the secondary drug screen on 2D cultures of all UPS cell lines.

**Table** **S9.** The sensitivity profiles of the five UPS cell lines to selected drugs and the associated genomic alterations identified in them.

**Table** **S10.** The complete results of the trametinib and infigratinib or doxorubicin combinations screen on 2D cultures of UPS cell lines.

**Methods S1.** xxx.
